# Can a humanoid social robot stimulate the interactivity of cognitively impaired elderly? A thorough study based on computer vision methods

**DOI:** 10.1007/s00371-021-02242-y

**Published:** 2021-07-30

**Authors:** Gauri Tulsulkar, Nidhi Mishra, Nadia Magnenat Thalmann, Hwee Er Lim, Mei Ping Lee, Siok Khoong Cheng

**Affiliations:** 1 Institute of Media Innovation, Nanyang Technological University, Singapore, Singapore; 2 MIRALab, University of Geneva, Geneva, Switzerland; 3 Goshen Consultancy Services Pte Ltd, Singapore, Singapore; 4 Bright Hill Evergreen Home, Singapore, Singapore

**Keywords:** Social Assistive Robotics (SAR), Robot companions, Social robots, Social intelligence for Robots, Human–humanoid interaction, Computer vision, Observational scales

## Abstract

Social Assistive Robotics is increasingly being used in care settings to provide psychosocial support and interventions for the elderly with cognitive impairments. Most of these social robots have provided timely stimuli to the elderly at home and in care centres, including keeping them active and boosting their mood. However, previous investigations have registered shortcomings in these robots, particularly in their ability to satisfy an essential human need: the need for companionship. Reports show that the elderly tend to lose interests in these social robots after the initial excitement as the novelty wears out and the monotonous familiarity becomes all too familiar. This paper presents our research facilitating conversations between a social humanoid robot, Nadine, and cognitively impaired elderly at a nursing home. We analysed the effectiveness of human–humanoid interactions between our robot and 14 elderly over 29 sessions. We used both objective tools (based on computer vision methods) and subjective tools (based on observational scales) to evaluate the recorded videos. Our findings showed that our subjects engaged positively with Nadine, suggesting that their interaction with the robot could improve their well-being by compensating for some of their emotional, cognitive, and psychosocial deficiencies. We detected emotions associated with cognitively impaired elderly during these interactions. This study could help understand the expectations of the elderly and the current limitations of Social Assistive Robots. Our research is aligned with all the ethical recommendations by the NTU Institutional Review Board.

## Introduction

Cognitively impaired elderly citizens in nursing homes require holistic care, including medical, nursing, physical and functional, psychosocial, and cognitive care. However, most institutions prioritise medical, nursing, and physical and functional care services over psychosocial and cognitive needs. It is common for the elderly in nursing homes to be lonely, socially isolated, bored, and lack activity engagement, resulting in other issues, such as concerning behaviours, adjustment difficulties, poor mood, and lack of motivation. Such psychological impact often affects the overall health and well-being of these residents.

Several research teams are currently working on projects for elderly self-sufficiency [1, 2], particularly on conversational agents design [3, 4]. Critical to building a coherent, proactive, and engaging conversational social robot is social dialogue exchange. An autonomous social humanoid robot with intelligent perceptual analyses provides better engaging interactions and could foster a personalised relationship with its user (5). Such a relationship could help address a user’s psychosocial and cognitive needs and reduce the issues (listed above) that increase the demand for care and human resources. Social robots could address workforce shortages and improve the engagement and participation levels and the quality of life of the elderly in nursing homes.

This research required extensive support from the care and therapy staff to boost declining interaction levels caused by communication, language, and cognitive obstacles. To implement such a robotic solution, we evaluated several interactions between professional therapists and the elderly. We identified appropriate and proactive behaviours that would stimulate the humanoid social robot to show empathy and interact meaningfully with cognitively impaired elderly. We then integrated these behaviours into competency descriptors and incorporated them into our humanoid social robot, Nadine [5].

We aimed primarily to stimulate residents of a nursing home through conversations and keep them engaged. We deployed Nadine to provide companionship and support to nursing home residents with cognitive impairment. The human–robot interaction (HRI) technology was used for the following purposes:

- *To promote engagement and stimulation using various stimuli:* Nadine should be able to interact with residents in nursing homes in multiple ways, including playing games (Bingo, karaoke), holding a conversation, showing images/videos, and playing songs.
- *To provide person-centred care through personalised interactions:* Based on face recognition, memory networks for conversations, and other AI techniques, Nadine should provide a personalised interaction service to each resident to help improve their memory, help them recall facts and emotions, and improve their quality of life in a nursing home.

We assessed Nadine’s efficacy based on residents’ willingness to communicate and engage with her and their moods during these interactions. We compared results from initial interactions to the changes in interaction over time to evaluate participants’ emotional states and quality of engagement. All interactive sessions were recorded, and a comprehensive understanding of the effects of Nadine was obtained using objective and subjective tools. The objective tools were based on computer vision techniques, such as Deep Neural Networks (DNNs), while the subjective tools relied on the Observed Emotion Rating Scale (OERS) [6] and Menorah Park Engagement Scale (MPES) [7]. OERS is an observational rating scale that grades the extent or duration of an affective state, such as pleasure, anger, anxiety/fear, sadness, and general alertness in the elderly. MPES is an observational rating scale that appraises 4 types of engagement: nonengagement (e.g. blank stare), self-engagement (e.g. fiddling with clothes), passive engagement (e.g. listening), and constructive engagement (e.g. actively handling objects).

The rest of the paper is organised as follows: Section 2 looks at related investigations using humanoid robots in nursing homes. We also touch on some humanoid robots providing support to the elderly with cognitive impairment. Section 3 discusses our research questions to be addressed. Section 4 explains our experimental setup of Nadine at the nursing home and the adaptation technique used. In Sect. 4.5, we outline the details of our data collection methods and provide our framework to analyse the data collected. Section 5 presents the results of the analysis of our experimental data. We address potential limitations and provide conclusions in Sect. 6. Finally, Sect. 7 discusses future plans.

## Literature Survey

Significant efforts have been aimed at improving the overall mental health of the elderly by providing them with robots for companionship. Robots, such as Pearl [8, 9], Silbot [10], Mero [11], Hobbit [12–14], Lio [15], Kompaï [3], TIAGo [16], RAMCIP [17], Matilda [18], and more provide the elderly with assistance or services, like fulfilling daily activities, that enable them to live in their own homes and communities safely and independently [19–24]. The elderly in nursing homes have been observed to engage more frequently with various stimuli, including life-like dolls, plush animals, building blocks, sorting tasks, puzzles, and robots [25].

Investigators have recognised that a participant’s choice of a robot is strongly motivated by its physicality. Researchers [26, 27] investigating the effects of the physical appearance of social robots have reported that embodiment plays a vital role in their interactivity. Social assistive-robot-based systems that can perform activities, play cognitive games, and socialise, can stimulate people’s cognitive, social, and physical conditions and constrain the deterioration of their cognitive state [19].

**Fig. 1:**
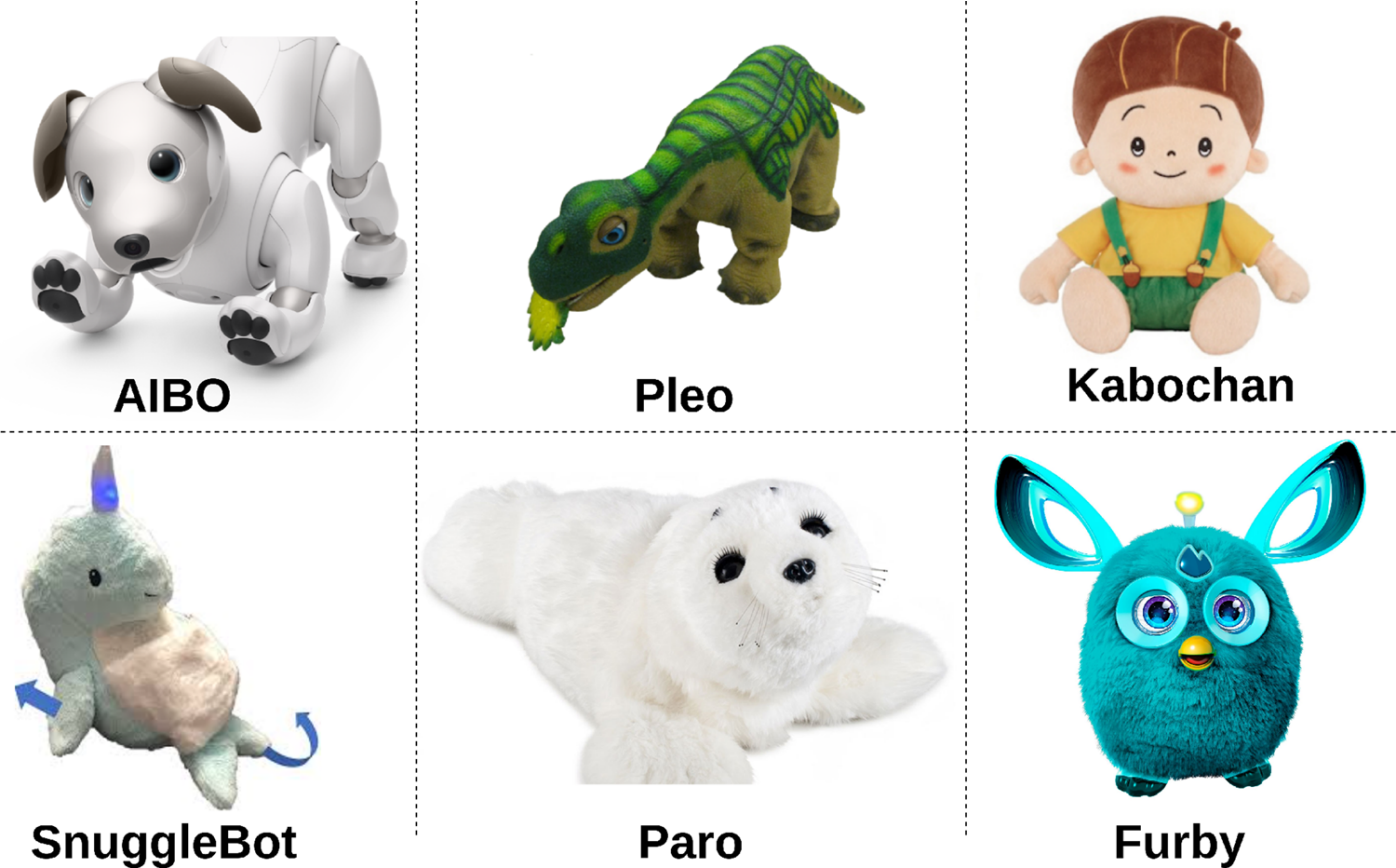
Animal-inspired Robots

Many animal-inspired robots, such as Paro [28–35], AIBO [36–40], iCat [41], Furby [42], NeCoRo [43], CuDDler [44], have been used to maintain interest in the elderly and have yielded positive social and psychological effects akin to animal-assisted therapy. However, creature-like robots, such as the hobbit robot, have eventually been treated as ordinary toys, rather than an aid, and have failed to become meaningful companions for the elderly [45]; these shortcomings in robot performance eventually frustrated residents.

The relationship between Paro robots and residents in care facilities revealed that Paro strengthened social ties among residents, with most residents establishing moderate to strong ties with the robot; it also decreases residents’ stress levels [28, 46–52].

While most creature-like social robots have been designed to provide companionship to the elderly, few have been explicitly designed to improve psychological well-being among this group of citizens. As robots, Paro and Aibo do not necessarily satisfy all the needs of the elderly, such as improving social interaction [53]. Pleo [54]), which was developed as a toy in the form of a baby dinosaur and can learn voice commands, sense specific food, and react to touch, has been used in homes for studies. Initially, participants were fascinated with the robot’s novelty, but they soon got fed up and did not interact with it regularly. Eventually, Pleo was only activated on special occasions. While Pleo was initially treated like a real animal with activities, such as petting and being named, it failed to inspire regular interactions and was treated as a regular toy over time.

Humanoid-like robots, such as Nao, Pepper, and Buddy, are also used to keep the elderly company [55, 56]. Research with Nao robots as physical exercise teachers and instructors has shown that they can guide exercises, deliver information to users, and provide motivation in the same way as human instructors [57]. Another study [58] demonstrated that interaction with Nao improved patients’ evaluated parameters. User expectations are directly related to robots’ appearances. We used Nadine for our study because she has a realistic-humanoid appearance and can hold engaging conversations with the elderly.

**Fig. 2:**
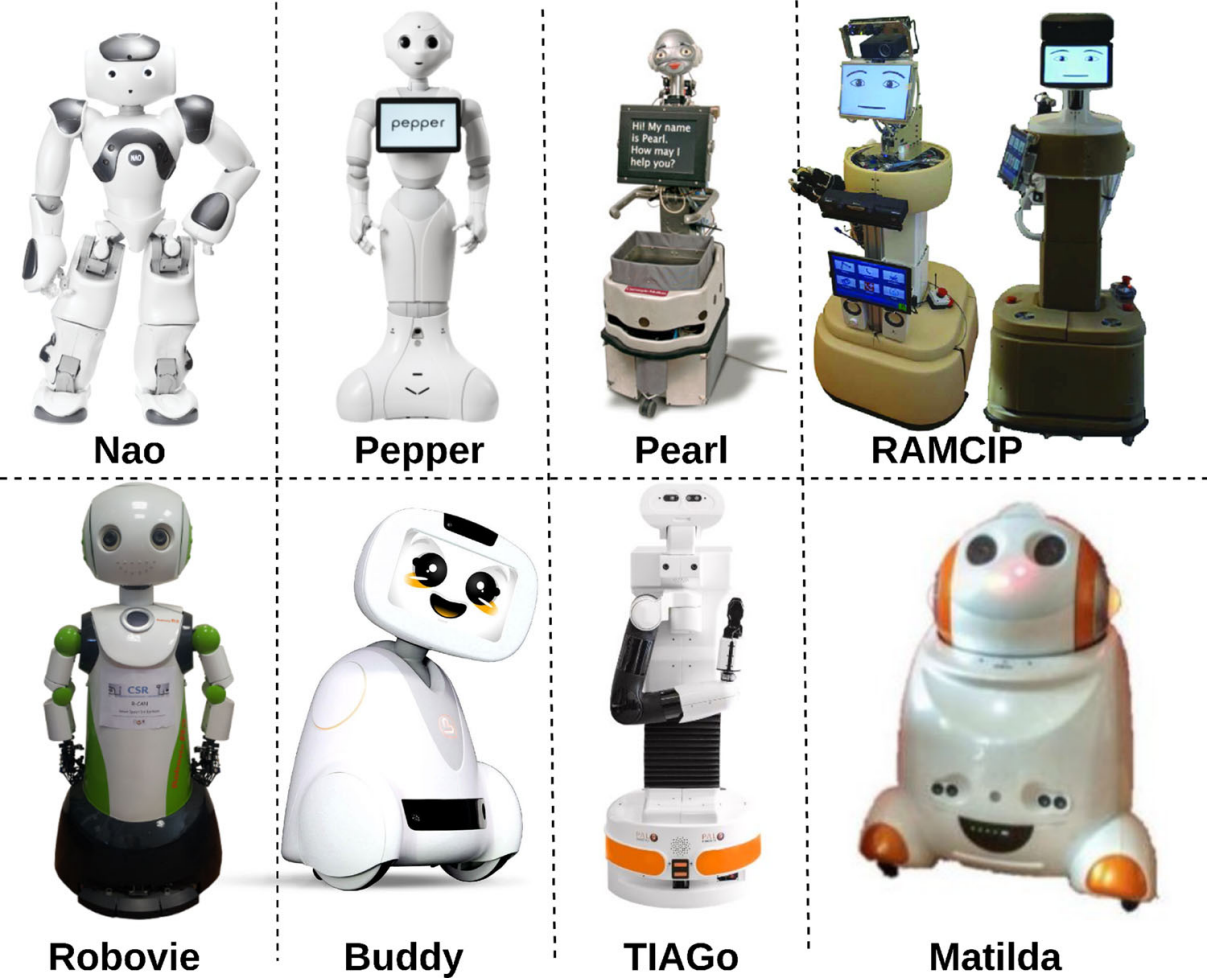
Human-inspired Robots

**Table 1:** Summary of the robots used for interaction with and companionship for the elderly, where (a) is Humanoid realistic appearance, (b) is Residents information, (c) is Empathy, (d) is Facial expressions, (e) is Gestures, (f) is The Internet of Things (IoT), (g) is Gazing, (h) is Face recognition, (i) is Languages spoken, (j) is Pro-active mode, (k) is Social Activity host, (l) is Number of participants, (m) is Computer Vision-based analysis, (n) is Personalisation, and (o) is Observational tool-based analysis

	(a)	(b)	(c)	(d)	(e)	(f)	(g)	(h)	(i)	(j)	(k)	(l)	(m)	(n)	(o)
[59] Pepper	X	X	$\checkmark$	X	$\checkmark$	$\checkmark$	$\checkmark$	N/A	$\checkmark$	N/A	$\checkmark$	11	X	N/A	X
[60] Kabochan	X	X	$\checkmark$	X	$\checkmark$	X	X	X	$\checkmark$	$\checkmark$	X	52	X	X	$\checkmark$
[61] Zora	X	N/A	$\checkmark$	X	$\checkmark$	$\checkmark$	$\checkmark$	N/A	$\checkmark$	$\checkmark$	$\checkmark$	60	X	N/A	$\checkmark$
[62] Zora	X	N/A	$\checkmark$	X	$\checkmark$	$\checkmark$	$\checkmark$	N/A	$\checkmark$	$\checkmark$	$\checkmark$	245	X	N/A	$\checkmark$
[63] Eva	X	$\checkmark$	$\checkmark$	X	$\checkmark$	$\checkmark$	$\checkmark$	$\checkmark$	$\checkmark$	$\checkmark$	$\checkmark$	9	X	$\checkmark$	$\checkmark$
[64] Multiple	X	$\checkmark$	$\checkmark$	$\checkmark$	X	$\checkmark$	$\checkmark$	$\checkmark$	$\checkmark$	$\checkmark$	$\checkmark$	13	X	$\checkmark$	N/A
[65] Ryan	X	$\checkmark$	$\checkmark$	$\checkmark$	$\checkmark$	$\checkmark$	$\checkmark$	$\checkmark$	$\checkmark$	$\checkmark$	$\checkmark$	6	X	$\checkmark$	X
[66] Sophie, Jack	X	$\checkmark$	$\checkmark$	X	X	$\checkmark$	$\checkmark$	$\checkmark$	$\checkmark$	$\checkmark$	$\checkmark$	139	X	X	$\checkmark$
[67] Nao	X	$\checkmark$	$\checkmark$	X	$\checkmark$	$\checkmark$	$\checkmark$	$\checkmark$	$\checkmark$	$\checkmark$	$\checkmark$	9	X	$\checkmark$	$\checkmark$
[18] Matilda	X	$\checkmark$	$\checkmark$	$\checkmark$	$\checkmark$	X	$\checkmark$	$\checkmark$	$\checkmark$	$\checkmark$	$\checkmark$	70	X	$\checkmark$	$\checkmark$
[68] Paro	X	X	$\checkmark$	X	$\checkmark$	X	$\checkmark$	X	X	X	X	14	X	X	X
[69] Joy for all	X	X	X	X	$\checkmark$	X	$\checkmark$	X	X	X	X	8	X	X	$\checkmark$
[70] Tangy	X	X	$\checkmark$	X	$\checkmark$	$\checkmark$	$\checkmark$	X	$\checkmark$	$\checkmark$	$\checkmark$	7	X	N/A	X
[71] Stevie	X	$\checkmark$	$\checkmark$	$\checkmark$	$\checkmark$	$\checkmark$	$\checkmark$	$\checkmark$	$\checkmark$	$\checkmark$	$\checkmark$	N/A	X	$\checkmark$	N/A
[72] Silbot	X	$\checkmark$	$\checkmark$	$\checkmark$	$\checkmark$	$\checkmark$	$\checkmark$	$\checkmark$	$\checkmark$	$\checkmark$	$\checkmark$	19	X	X	$\checkmark$
[73] Pleo	X	X	$\checkmark$	X	$\checkmark$	X	X	X	X	X	X	14	X	X	$\checkmark$
[74] SnuggleBot	X	X	X	X	$\checkmark$	X	X	X	X	X	X	N/A	N/A	X	N/A
[75] Robovie	X	X	$\checkmark$	$\checkmark$	$\checkmark$	X	$\checkmark$	$\checkmark$	$\checkmark$	$\checkmark$	$\checkmark$	11	X	X	X
[41] iCat	X	$\checkmark$	$\checkmark$	$\checkmark$	$\checkmark$	$\checkmark$	$\checkmark$	$\checkmark$	$\checkmark$	$\checkmark$	$\checkmark$	47	N/A	$\checkmark$	$\checkmark$
[56] Buddy	X	$\checkmark$	$\checkmark$	$\checkmark$	$\checkmark$	$\checkmark$	$\checkmark$	$\checkmark$	$\checkmark$	$\checkmark$	X	40	N/A	$\checkmark$	N/A
[8] Pearl	X	$\checkmark$	$\checkmark$	X	$\checkmark$	$\checkmark$	$\checkmark$	N/A	$\checkmark$	$\checkmark$	N/A	N/A	N/A	$\checkmark$	N/A
[76] virtual CommU	X	X	$\checkmark$	X	$\checkmark$	X	$\checkmark$	X	$\checkmark$	$\checkmark$	X	40	X	X	X
[76] CommU	X	X	$\checkmark$	X	$\checkmark$	X	$\checkmark$	X	$\checkmark$	$\checkmark$	X	40	X	X	X
Nadine	$\checkmark$	$\checkmark$	$\checkmark$	$\checkmark$	$\checkmark$	$\checkmark$	$\checkmark$	$\checkmark$	$\checkmark$	$\checkmark$	$\checkmark$	14	$\checkmark$	$\checkmark$	$\checkmark$

One study [77] explained the importance of advancing the understanding of HRI by using social robots to address attention issues in the elderly. The elderly benefit from communicating with robots [78]. Research with the Kabochan speaking robot [60, 79] demonstrated an increase in resident-robot engagement over time in a group, but direct engagement was not assessed. Having a physical body and a system capable of handling a conversation flow in a robot is advantageous in promoting high engagement of the elderly [76]. These findings of the study using a virtual and a physical robot CommU provide a new insight into the development of dialogue systems for robots in assisting elderly by maintaining a better mental health.

Ethnographic studies in which a humanoid social robot, Robovie, interacted with residents in an elderly care centre [75, 80] suggested that the robot was well accepted. However, Robovie’s behaviour was remotely operated to act as a conversational partner through basic dialogues that only included greetings and questions, particularly questions about hobbies. Robovie as a companion was appreciated, and the elderly even took walks with it. However, no significant improvements in residents’ cognitive abilities have been observed when the elderly have walked with/without Robovie or enjoyed its company [81] because no interaction has been noted during those activities.

In a study evaluating the Kompaï robot [82] designed by Robosoft [83] two elderly participants interacted with Kompaï for almost an hour. Significant sensory data, such as audio data, RGB-D data, thermal images, and data from the skeleton tracker, were recorded from the multisensory system to gain contextual insight into the users’ interactions, needs, and reactions. Analyses showed that these reactions were positive, and the two elderly found the robot to be beneficial [3] . However, this study was performed for a limited time, and the two users did not constitute enough sample to advance a general conclusion. Other studies have since also supported these results, through [16, 20, 84].

In one study, the Pepper Robot [59] better understood multidomain interventions vis-à-vis social facilitation with the elderly. At the end of the inquiry, participants showed strong emotions when letting go of the robot, indicating the importance of the social bond they had created with it. The Pepper robot has also been used in cases of schizophrenia and dementia for recreational purposes [85, 86].

Healthcare professionals have a favourable view of the effect of the Zora robot [62]. However, negative impacts resulting from the use of robots for elderly care also exist [87]. The elderly consider social robots to only aid recreational activities. The impact of Zora on care personnel and elderly participants in care services was evaluated previously for 10 weeks, with findings fluctuating between negative and positive feedbacks [61].

Monitoring and managing emergencies and helping with robotic support have been widely accepted [88, 89], whereas tasks involving the direct interaction between a person and a robot are not yet recognised.

Individual adaptations of robots for dementia or cognitively impaired persons, like verbal reminders and personal adaptation, are essential. A mixed method showed the positive outcomes of several aspects, such as apathy, quality of life, and cognitive state, of residents’ dispositions [90].

## Conclusions of the SAR, choice of humanoid robot and research questions

We incorporated the competencies presented in table 1 into our robot to create more meaningful interactions missing in earlier studies, as discussed below. For appearance, we indicated whether the robot is human-like. We aimed to assess if a humanoid robot, such as Nadine with its human-like features, could emote natural human communication, as reported in [91] and satisfy the innate need for human companionship among the elderly. Nadine can make facial expressions, respond with gestures, and make eye contact with the elderly, something other robots have not been able to do in the past. Studies have shown that the interaction with face and arm movements is stimulating and could arouse curiosity and interest [10].

For functionalities, our study was based on speech, vision, empathy, memory, and The Internet of Things (IoT). We analysed the following:

- The robot’s ability to speak and understand speech; if it could talk, is the robot multilingual? [92] claims robots need to communicate with the elderly to provide care and companionship;
- Different aspects of the robot’s vision-based capabilities for functionalities. We assess whether the robot can detect faces, recognise them, and understand the environment to gaze at the elderly and understand their facial emotions;
- Nadine’s conversational and empathetic capabilities vis-à-vis other robots. Nadine is a conversational, empathetic robot, and we compare this capability with other robots [17, 93]. Nadine is a conversational, empathetic robot, and we compare this capability with other robots.
- If previous robots were capable of engaging the elderly using memory. The ability to give reminders and remember past conversations to initiate and sustain personalised conversations is crucial to a robot’s ability to provide companionship to the elderly [92]). Having a long-term memory would allow Nadine to create a high-level behavioural system, enabling her to interact with the elderly in richer forms. Nadine already could use previous memories of places, people, objects, or performed behaviours to enhance interactions and establish long-term relationships [94].
- If previously studied robots could control and interact with the devices around them, such as TV, speakers, and temperature control. IoT is an emerging vision that brings together pervasive sensors and objects with robotic and autonomous systems [95].

Apart from making conversation, we intended to find out what other activities these robots could host. We also classified these activities based on the study environment and the number of participants. Furthermore, we built our classification around the analysis method used for the statistical results in previous studies, specifically, AI- or ML-enabled. We also inquired if the studies used observational tools, such as MPES and OERS, to analyse users’ engagement.

To reap the benefits of SARs for healthcare, key stakeholders, including healthcare professionals, therapy staff, and managers in institutional settings, must become essential role players in the eventual acceptance of robot technology [61]. Our contribution is to study several interactions between professional therapists and the elderly. These interactions are defined as appropriate behaviour models required for our robot to best perform at nursing homes. In our study, we considered the following holistic parameters:

- Personalised conversation using face recognition and long-term memory;
- Multilingual conversation with personalized speech;
- Computer vision and deep learning-based methods to quantify and gauge emotions;
- Nadine being a realistic humanoid robot to provide a human-like presence and companionship.

These parameters deduced from table 1 were not taken into account by previous investigations. We believe that our study could be a step forward in introducing a humanoid social robot with a human-like appearance and characteristics that mimic human behaviour, emotion recognition, and gesture synthesis. We were the first to use deep learning-based model parameters to verify and validate results obtained in data analysis. Our evaluation method incorporates video analysis and the use of observational tools, like OERS and MPES, to determine the holistic effect of the conversation sessions with Nadine as a companion for the elderly.

Robots have many advantages, including providing constant help and ensuring they offer the same quality and consistency without discrimination. They are also less likely to make mistakes. However, they, undoubtedly, lack personality, human empathy and naturalness, and a human personal approach—for the time being. The current research aimed to address the possible acceptance and limitations of the robotic technology used in the broad area of HRI up to now. In this regard, we intended to answer the following research questions:

- Does a social robot that can initiate contact and have personal information of residents encourage interaction, participation and engagement? (the residents sought to interact with Nadine without staff, calling Nadine by name, showing “affection” to Nadine, looking at Nadine or calling Nadine for numbers).
- Does the presence of a social robot increase social interactivity (connect with the residents, increase smiling, laughing and conversations) amongst residents within the environment?
- Does the presence of a social robot reduce the amount of time/attention from care staff to attend to the negative emotional events of the residents, e.g. quarrelling amongst (cognitively impaired elderly), repeated calling for staff, apathetic behaviour and low engagement? (e.g. positive behavioural responses, like paying attention and talking to Nadine, curiously observing Nadine instead of indifference/“stoning”).

**Fig. 3:**
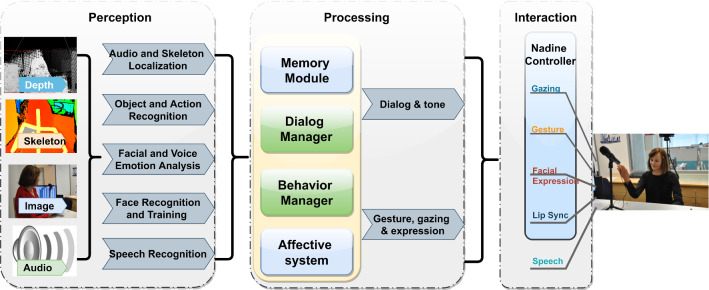
Nadine Robot’s Architecture

## Experimental setup

We used Nadine, a socially intelligent, realistic humanoid robot with natural-like skin, hair, and appearance, for our experiments. She was placed at the centre of an elderly home ward for this experiment. Fourteen residents participated in 29 one-to-one interaction sessions with the help of the care staff. To understand the overall results at the end of the study, Nadine was left running for three hours each morning for six days thereafter without the care staff to observe residents’ initiative. During interaction sessions, residents could interact with Nadine; she initiated conversations when she observed residents being inactive/passive for a long time.

In order to get a holistic idea of the effect of Nadine’s interaction sessions with the residents, we placed five different cameras at different angles to record the one-to-one interaction sessions. Complete footage from all available cameras was analysed and compared without compressing them into single average sessions.

Using the same video footage as the one analysed by computer vision methods, we conducted an observational analysis to validate our findings. Thus, Nadine’s potential to incite the engagement of residents in the activity and its effect on their emotional state were studied.

### Ethical protocol

Our research was reviewed by the Research Integrity & Ethics office and approved by the Institutional Review Board of NTU under the application number IRB-2020-09-056.

Participation in our study was entirely voluntary. It was the participant’s choice of whether to participate or not. Participants had the right to refuse to participate or withdraw from the study, even if the participant had agreed to participate earlier. All participants were given pertinent information (according to their level of understanding and in the language that they understand) to make an informed decision about participating in the study. They signed a consent form if they agreed to participate in the study. For non-English speaking participants, an interpreter fluent in both English and the participant’s spoken language was used to help in the consent process. A representative of the NTU research team, a BHEH medical social worker and a Certified Dementia Practitioner from Goshen Consultancy Services Pte Ltd conducted the process of seeking consent from the residents using a new up to standard NTU-IRB Consent form to convey the complete information of the study to the participants in layman terms to help the participants make an informed decision. BHEH medical social worker and Goshen Consultancy Services Pte Ltd who are Certified Dementia Practitioner helped us select the right participants.

### Covid-19-related safety and precautions

In considering the ethical issues in various applications of robotics in elderly care, we have observed and critically emphasized on the safety protocols and safe distancing measures for Covid-19. BHEH maintained and followed the guidelines given by the Ministry of Health. As older people are more vulnerable to Covid infections, utmost care in personal hygiene and safe distancing in line with the rules and regulations set by the Ministry of Health, Singapore, were undertaken. The NTU research team underwent temperature monitoring and safety check-in before every experiment. Research team maintained the one-meter distance with the elderly and made no physical contact with them. NTU research team visits were limited to software and hardware maintenance of the Nadine robot. Masks were worn by all care-staff and the research team at all times. Sanitizers and wet wipes were placed all across the ward room for disinfecting the equipment, such as microphones and the surfaces touched by the residents and the team. Each surface and items were disinfected before and after each experiment. The experiments and the visits to the nursing home commenced only when it was safe and duly advised by the Ministry of Health, Singapore.

### Architecture

Nadine has 27 degrees of freedom (DOF), enabling her to make facial movements and gesticulate effectively, as documented in [96] and [97].

Figure 5 shows Nadine’s architecture, as developed by [5]; it consists of three layers: perception, processing, and interaction. Nadine receives audio and visual stimuli from microphones, 3D cameras and web cameras to perceive user characteristics and her environment, which are then sent to the processing layer. The processing layer is the core module of Nadine that receives all results about the environment from the perception layer and acts upon them. This layer includes various submodules, such as dialogue processing (chatbot), affective system (emotions, personality, mood), and Nadine’s memory of previous encounters with users. The action/interaction layer consists of a dedicated robot controller, including emotion expression, lip synchronisation, and gaze generation.

Nadine can recognise people she has met before and the faces she is trained to identify and engage with in an ongoing flowing conversation. She can also help people read text, show images, put on Skype sessions, and respond to emails. Nadine can be considered part of the human-assistive technology [98], as she can assist people over a continuous period without breaks; she has previously worked at different places that required her to work for long hours [99].

### Adaptation

Nadine supports an adaption based on the Nir Eyal model of lifecycle hooks presented by Eyal [100]. Any habit starts with an external trigger, followed by usage, and the usage stimulates more usage, an advantage the elderly can gain from the robot. Nadine’s perception engine helps understand the habitual lifecycle of the elderly, prompting an internal trigger in the elderly to use the robot further. She carefully records residents’ data and gives intelligent, actionable inferences on their likes and dislikes. Empathy mapping involves actions based on the inferences, which further strengthens residents’ attachment to the robot companion; this is the lifecycle hook. We aimed to determine how the lifecycle hook of the elderly’s behaviour improves interaction as the resident starts investing time with the robot companion and ensures that an internal trigger is set off to prompt the elderly to use the companion. Studies have proven that collaborative care models have improved the health of the elderly. The objective of this research was to see Nadine’s impact and improve her Empathy-AI mapping capabilities.

[101] and [102] reported that most robots designed for the elderly do not fulfil the needs and requirements to perform their best. For Nadine to perform her best at nursing homes, we updated some previous models and developed new modules recommended in an earlier study [103]. The modules that were updated/expanded for Nadine as a caretaker at a nursing home are as follows:

- Show images, songs and videos
- Proactive module
- Recognise residents
- Update Nadine’s Personal Memory and Response
- Update in Affective System—Emotion Engine
- Update in speech tone and speed

#### Show images, songs and videos

We created an interface that plays songs or videos or shows images of residents’ choice to enable them to interact with Nadine. The module allows residents to play songs and videos and show images if these items are available in the database; the nursing home staff created the database based on the likes and usual requests from residents. Nadine played the requested item triggered by the keywords on the database, and this item was displayed on the external touch screen.

**Fig. 4:**
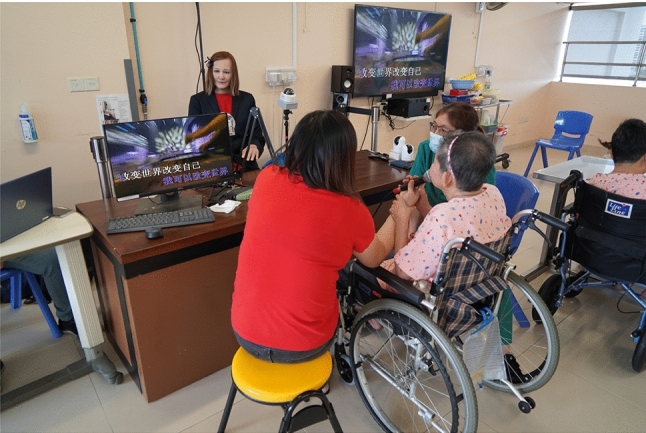
Nadine playing videos for residents

#### Proactive module

In social and healthcare assistive robotics, proactiveness is highly desirable in robots that serve as companions or the care staff. Initiating a conversation is a proactive task. Nadine can respond to questions and keep up the conversation but cannot proactively initiate a conversation. We developed and deployed an uncomplicated proactive module that enables Nadine to start a conversation proactively to solve this problem. The goal is for Nadine to be able to start a conversation and continue to keep residents engaged in it.

To create this module, we used the BDI framework proposed in [104]. We also integrated a state-of-the-art gaze tracking model in Nadine, in which the coordinates of the point of gaze of the subject are estimated using the model proposed in [105]. This point is calibrated with the Kinect depth map as a reference to translate to real-world coordinates of the gaze point. A threshold that helps Nadine recognise when a resident is quiet or passive for a very long time and then triggers her to initiate a conversation from her proactive module was applied.

#### Recognise the residents

Nadine uses the identity of a person to customise her responses and behaviour. For face recognition, a web camera attached to Nadine focuses on the person currently talking to her. She uses a deep neural network based on solutions in [106], which can detect faces and identify them. From a training perspective, only 10 images of a person are required to achieve a good performance. Ten images of each resident were acquired and used to train Nadine to identify them.

During the interaction, the face is passed through the trained model to identify the resident using the web camera stream; this information is critical to Nadine’s operation. A user identity or name acts as an input to other submodules to change verbal and nonverbal behaviours. For example, during interactions, verbal responses can include the name of the resident. Moreover, based on the person’s identity, the memory submodule loads different memories, including previous conversations with the user, to trigger appropriate verbal and nonverbal responses, depending on the context of the conversation. The affective module also uses this information to react to emotion and mood changes if necessary.

#### Update Nadine’s personal memory and response

To better suit residents, we updated the questions and responses in Nadine’s personal memory. Her responses to the residents should rather be enriching than chatbot-based. Based on our study with therapists at nursing homes, we understand that Nadine’s response should be positive and not end with a statement. She should show curiosity, and her answers should be in a manner that invokes reactions from the residents and keeps them involved in the conversation. For this, we updated Nadine’s personal memory and responses in the chatbot.

#### Update in affective system—emotion engine

Any individual can be characterised by their personality and emotions. Subconsciously, people convey different messages through their display of emotions. Changes in emotion can occur in the form of facial expressions, change in tone and pitch of speech, and gestures. As a social humanoid robot, Nadine has an emotion engine that controls her sensations, personality, and mood during interactions, enabling her to perceive the situation (user and environment) and adjust her emotions and behaviour accordingly. As a result, Nadine can generate different emotions, including pleasure, arousal, and dominance.

For Nadine to perform best in nursing homes, she needs to appear patient and show no negativity or anger. Therefore, Nadine should exhibit a positive temperament only. A configuration file was set to different parameters that allow Nadine to stay positive and behave accordingly, even when residents are frustrated, angry, or upset with her.

#### Update in speech’s tone and speed

Another vital aspect for Nadine’s optimal functioning is to reveal positive emotions in her speech synthesis output, mainly by changing her speech’s pitch, tone, and speed and including modulations. We modified the speech synthesiser to adapt speech output so that Nadine speaks slower and louder and in a low tone to make it easier for residents to understand her.

### Data analysis

Using the analytical procedures provided below, we obtained the data for every video of Nadine’s interactions with all residents and analysed them using statistical methods to get meaningful comparisons.

**Fig. 5:**
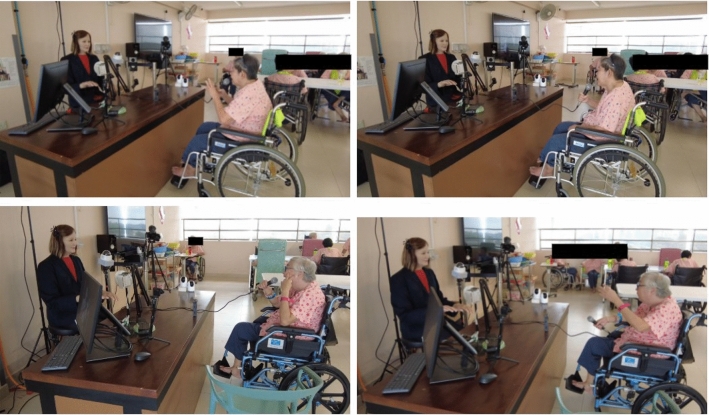
Residents interacting with Nadine in one-to-one sessions

#### Video data analysis using the computer vision-based method

Analysing emotional facial expressions is essential to understanding feelings and determining whether the conversations are enjoyable. Facial Expression Recognition has promising applications in human-computer interaction and mental health assessment. In order to get a holistic idea of the effect of Nadine’s interaction sessions with residents at the Nursing home, we placed five different cameras at various angles to record the sessions and analysed the videos of 11 residents.

Because our research focuses on the engagement of the elderly in one-to-one interactions, we analysed only the two cameras placed at angles that directly focused on the faces of the elderly. Five different cameras were placed at different angles throughout the room to capture the elderly as a group from different angles. However, the footages obtained from the two specific cameras angled directly at the elderly allowed us to get a clear view of the expressions of the individual elderly and their movements and gestures while conversing with Nadine on a one-to-one basis. On the videos obtained, we used objective tools based on computer vision techniques to evaluate the emotional and physical states of the elderly to understand their engagement in the conversations. Emotional cues, such as positive, negative, and neutral, found through facial expressions were used to determine the interest levels and engagement of the elderly. The following five evaluation metrics were studied:

- *Positive Emotional engagement*, which reflects the positive effect of the conversations with Nadine on the residents;
- *Negative Emotional engagement*, which reflects the negative effect of the conversations with Nadine on the residents;
- *Neutral Emotional engagement*, which reflects the neutral effect of the conversations with Nadine on the residents;
- *Movement*, which reflects Nadine’s effect on the physical movement of the elderly during the sessions;
- *Activity*, which reflects Nadine’s effect on the activity of the elderly during the sessions.

To provide a quantitative analysis of these five metrics, we exploited the advantages of DNNs in efficient video processing. Notably, we applied four different networks: a face detector^1^, an expression recogniser, an action detector^2^ and an optical flow estimator [107]. The face detector was used to estimate the locations of faces in any given frame of the videos. Moreover, because all the residents were in wheelchairs, their relative locations were inferred based on their facial locations. Only the faces of the elderly were detected, particularly as the nursing care staff were wearing medical masks throughout the sessions with Nadine. We adopted the Dlib library with its pre-trained convolutional neural network (CNN) to implement the face detector.

The expression recogniser categorised the expressions of detected faces. We considered eight expressions: Neutral, Happy, Sad, Surprise, Fear, Disgust, Anger, and Contempt. To this end, the ResNet-50 [108] was used, with the CNN architecture as the backbone, to categorise expressions. The recogniser was trained on the largest in-the-wild facial expressions dataset AffectNet [109] with about 320K (excluding None, Uncertain and Non-Face categories) images till the network was converged. In order to disentangle emotions during sessions, the expressions were grouped into 3 categories: Positive, Negative, and Neutral. The positive category consisted of Happy and Surprise, the negative of Sad, Fear, Disgust, Anger, and Contempt, and the neutral solely of the neutral expressions of the elderly.

The action detector measured the motions and actions of detected faces, generating action proposals, which are the locations and confidences of detecting an action. We implemented the action detector based on the pre-trained temporal segment network [110] provided in the MMAction2 library. The action detector informed us of the movement of the elderly during the session, as the face detector detected only their faces.

The optical flow estimator monitored moving targets. We estimated dense optical flow via the recurrent all-pairs field transformation network. For an arbitrary region in each frame during movement, the average magnitude of the estimated optical flow in the region was used to measure the intensity of movement of the elderly during the sessions.

The above DNNs were useful in spontaneous facial expression recognition, Activity and Movement detection in the continuous video stream of every resident. The quantitative measures of *Emotional engagement* and *Movement* were as follows:

*Positive Emotional Engagement* of residents was defined by the following equation using their positive expressions in every frame, where $s_t$ and $sur_t$ are the confidences of detecting Smile and Surprise expressions, respectively:

1 $$\begin{aligned}&\mathrm{smile} = \frac{1}{{L}}\sum \limits _{l = 1}^L {\frac{1}{{{n_l}}} \sum \limits _{t = 1}^{{n_l}} {{s_t}}} \end{aligned}$$

2 $$\begin{aligned}&\mathrm{surprise} = \frac{1}{{L}}\sum \limits _{l = 1}^L {\frac{1}{{{n_l}}} \sum \limits _{t = 1}^{{n_l}} {{sur_t}}} \end{aligned}$$

*Negative Emotional Engagement* of residents was defined by the following equation using their negative expressions in every frame, where $sad_t$, $f_t$, $disg_t$, $ang_t$ and $con_t$ are the confidences of detecting Sadness, Fear, Disgust, Anger, and Contempt, respectively:

3 $$\begin{aligned}&\mathrm{sadness} = \frac{1}{{L}}\sum \limits _{l = 1}^L {\frac{1}{{{n_l}}} \sum \limits _{t = 1}^{{n_l}} {{sad_t}}} \end{aligned}$$

4 $$\begin{aligned}&\mathrm{fear} = \frac{1}{{L}}\sum \limits _{l = 1}^L {\frac{1}{{{n_l}}} \sum \limits _{t = 1}^{{n_l}} {{f_t}}} \end{aligned}$$

5 $$\begin{aligned}&\mathrm{disgust} = \frac{1}{{L}}\sum \limits _{l = 1}^L {\frac{1}{{{n_l}}} \sum \limits _{t = 1}^{{n_l}} {{disg_t}}} \end{aligned}$$

6 $$\begin{aligned}&\mathrm{anger} = \frac{1}{{L}}\sum \limits _{l = 1}^L {\frac{1}{{{n_l}}} \sum \limits _{t = 1}^{{n_l}} {{ang_t}}} \end{aligned}$$

7 $$\begin{aligned}&\mathrm{contempt} = \frac{1}{{L}}\sum \limits _{l = 1}^L {\frac{1}{{{n_l}}} \sum \limits _{t = 1}^{{n_l}} {{con_t}} } \end{aligned}$$

For *Activity* and the *Movement*, we used the activity detection and the optical flow estimator, where $o_t$ and $d_t$ are the average magnitude of optical flow and the confidence of detecting activity, respectively, as follows:

8 $$\begin{aligned}&{ activity} = \frac{1}{{L}}\sum \limits _{l = 1}^L {\frac{1}{{{n_l}}} \sum \limits _{t = 1}^{{n_l}} {{o_t}}} \end{aligned}$$

9 $$\begin{aligned}&{ body}{\_}{} { movement} = \frac{1}{{L}}\sum \limits _{l = 1}^L {\frac{1}{{{n_l}}}\sum \limits _{t = 1}^{{n_l}} {{d_t}}} \end{aligned}$$

#### Video data analysis using observational tool

Residents were observed during their interactions with Nadine using video recording with the following tools:

- Observed Emotion Rating Scale (OERS) to determine the emotions expressed by the residents and
- Menorah Park Engagement Scale (MPES) to determine the level of engagement of residents.

The OERS and MPES observation data were collected over 4 sessions, with each session lasting from 8-25 mins. A summary of the 10 case studies was compiled to capture the changes and progress of residents. This summary was used to validate the results and findings of the observational data and qualitatively evaluate residents’ progress using a 3-domain conceptual framework [111].

The 3-domain conceptual framework (3D Framework) was developed to evaluate the impact of the one-to-one interaction with Nadine. This holistic framework that includes the psychosocial, emotional, and cognitive domains aimed to explain the impact of the one-to-one resident–Nadine interaction through the various domains and dimensions. The framework is conceptually organised in the following manner:

- *Psychosocial Domain: * This domain includes emotions, personality, self-esteem, and relationships that impacted empathy-related behaviours that residents demonstrated during interaction with Nadine.
  - Empathy-related Behaviours
- *Emotional Domain: * This includes the feelings and emotions that residents demonstrated during interaction with Nadine.
  - Emotional Expression
- *Cognitive Domain: * This includes the mental processes, thinking, learning, and understanding that residents demonstrated during interaction with Nadine.
  - Communication and Language Abilities
  - Orientation, Attention, and Memory

## Results

### Computer vision method

In order to understand the emotional reactions of the residents when interacting with Nadine, a repeated-measure ANOVA was conducted, revealing significant differences in the emotions experienced by the residents $(F (1, 316) = 1818.306, \textit{p} > .001)$. The levels of various emotions can be seen in Fig. 7

**Fig. 6:**
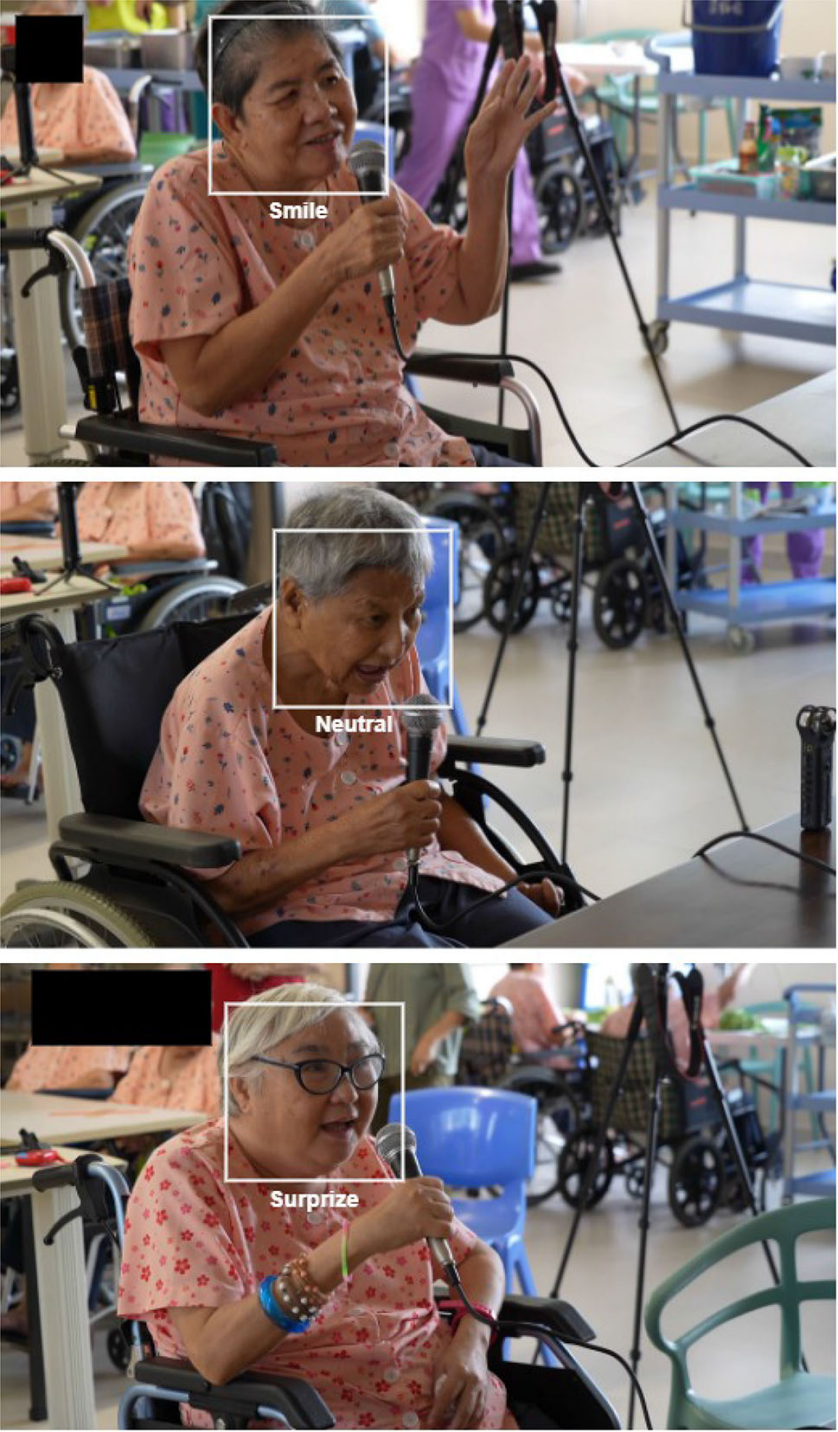
Different emotions recognised during one-to-one sessions

**Fig. 7:**
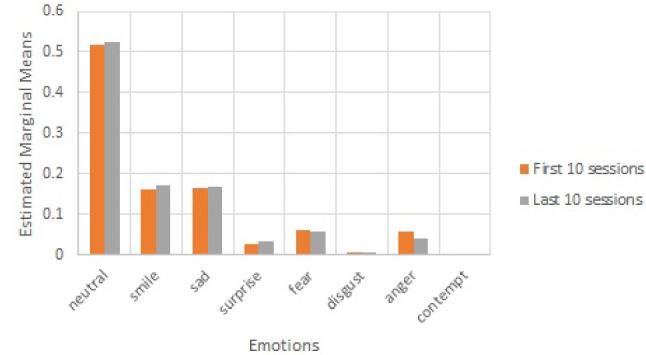
Means of different emotional expression

**Fig. 8:**
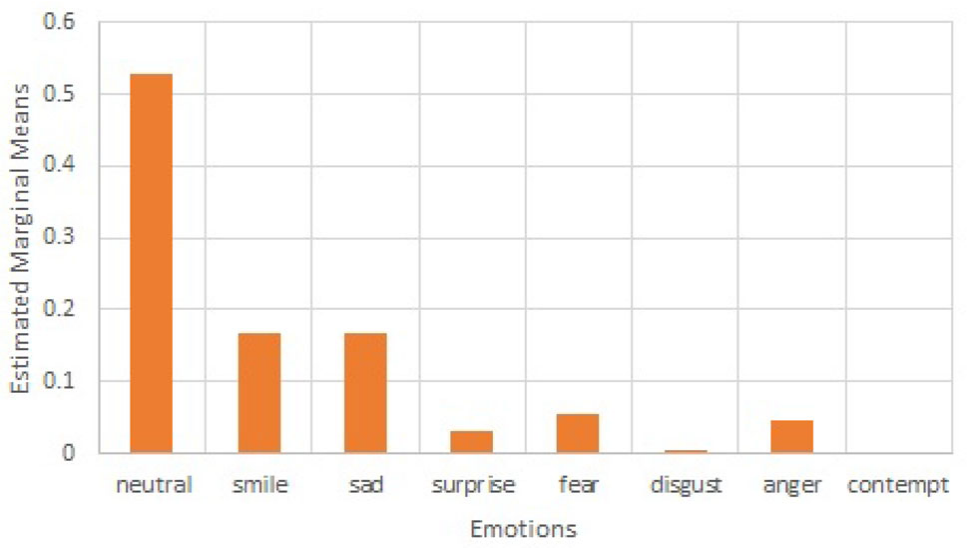
Comparing emotional patterns between the first and the last 10 sessions

Emotional patterns between the first ten and the last ten sessions were not different, as indicated by the absence of an interaction effect in the mixed ANOVA $(F (7, 1442) = .834, \textit{p} = .559)$ and also observable in Fig. 8.

In order to determine how Nadine’s presence affected the need for the activities of the care staff, the frequency of their visits was used as a predictor in linear regression, with the optical flow as the criterion. The model was significant $(F (1, 333) = 7.959, p = .005, \beta = .153)$, indicating that the activity of the care staff increased with Nadine’s presence over time. To determine the impact of Nadine’s presence on residents’ activity, linear regression with the frequency of their visits as the predictor and the body score as the criterion was performed. There were no significant connections between the two $(F (1, 335) = .589, p = .443)$. The mean number of visits was 14.25, with a standard deviation of 5.28. The frequency of visits for each of the residents can be seen in Table 2.

**Table 2:** Frequency of visits for each resident

Participant	Frequency	Body Score
1	20	0.17850973219
2	10	0.18772688969
3	20	0.19229858410
4	10	0.19545845097
5	12	0.20085960005
6	5	0.21649030833
7	22	0.20298815893
8	20	0.19874696114
9	11	0.22216335714
10	8	0.19246970280
11	11	0.20890980764
12	10	0.21042497391
13	8	0.20599271300
14	9	0.19257581925

The relationship between the number of visits and various emotions is presented in Table 3. People who interacted with the robot more showed more neutral expressions, less fear, and smiled less. The other correlations were not significant at the $\alpha = .05$ level.

**Table 3:** The correlations between various emotions and the frequency of visiting the robot

Emotions	Pearson Correlation	Sig. (2-tailed)
Neutral	.132	0.015
Smile	−.117	0.032
Sad	0.003	0.957
Surprise	−0.004	0.939
Fear	−.402	0.000
Disgust	0.069	0.208
Anger	0.074	0.175
Contempt	0.073	0.180
Body Score	−0.093	0.088
Optical Flow	0.104	0.058

The average duration of the conversations was 909 seconds (15 minutes and 9 seconds), with the standard correlation of 0.515. The serial number of the session successfully predicted the conversation length $(F (1, 335) = 18.610, \textit{p} < .001, \beta = .229)$ which increased over time. . Conversation length correlated negatively with smile and positively with neutral at the $\alpha = .05$ level (Table 4).

**Fig. 9:**
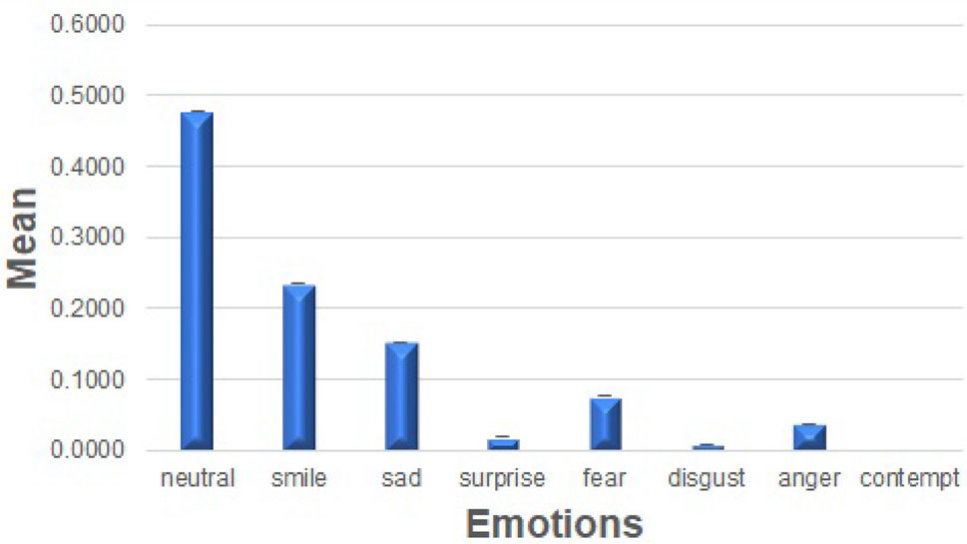
Emotion expression during open sessions

The data from the observation of video material of the two-week open session indicate that participants mostly felt positive emotions when interacting with Nadine; their predominant emotions were neutral and happiness (as shown in Fig. 9). Furthermore, participants never or very rarely showed disgust, contempt, fear, anger, and surprise; they showed a moderate amount of sadness. Pearson’s correlations revealed no difference in emotions based on the amount of time the residents spent with Nadine (*r* values ranging from .011 to .205, *p* values ranging from .174 to .943).

**Table 4:** The correlations between various emotions and the length of conversation

Emotion	Pearson Correlation	Sig. (2-tailed)
Neutral	0.067	0.218
Smile	.116	0.033
Sad	− .112	0.040
Surprise	− 0.041	0.455
Fear	− 0.027	0.628
Disgust	0.018	0.742
Anger	− 0.049	0.371
Contempt	− 0.005	0.931
Body Score	− 0.035	0.519
Optical Flow	0.036	0.508

### Observational Tools

OERS and MPES observational data showed positive changes in residents’ emotional state and engagement level over the 29 sessions of 1-to-1 interactions with Nadine.

**Fig. 10:**
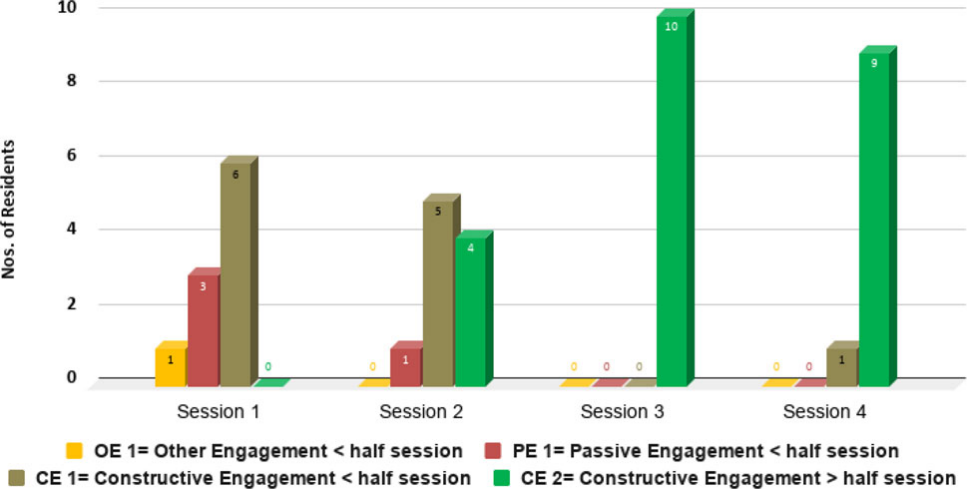
MPES Summary. Session one denotes the initial 10 sessions; Session two shows the subsequent 4 sessions; Session three indicates the next 5 sessions; Session four represents the final 10 sessions

Using the MPES tool, we observed residents’ engagement levels at baseline. The first 10 sessions yielded 10% Other Engagement (OE1), 30% Passive Engagement (PE1) and 60% Constructive Engagement (CE1). The engagement levels then steadily improved, as seen in the shift towards CE in the subsequent 9 sessions (100% CEu2) and final 10 (90% CE2 and 10% CE1). The MPES tool also showed improvements in residents’ engagement time, from less than half of the session at baseline to more than half of the session towards the end of the program, as shown in Fig. 10. In general, residents demonstrated improvement in the quality and duration of their engagement over the interaction programme with Nadine.

**Fig. 11:**
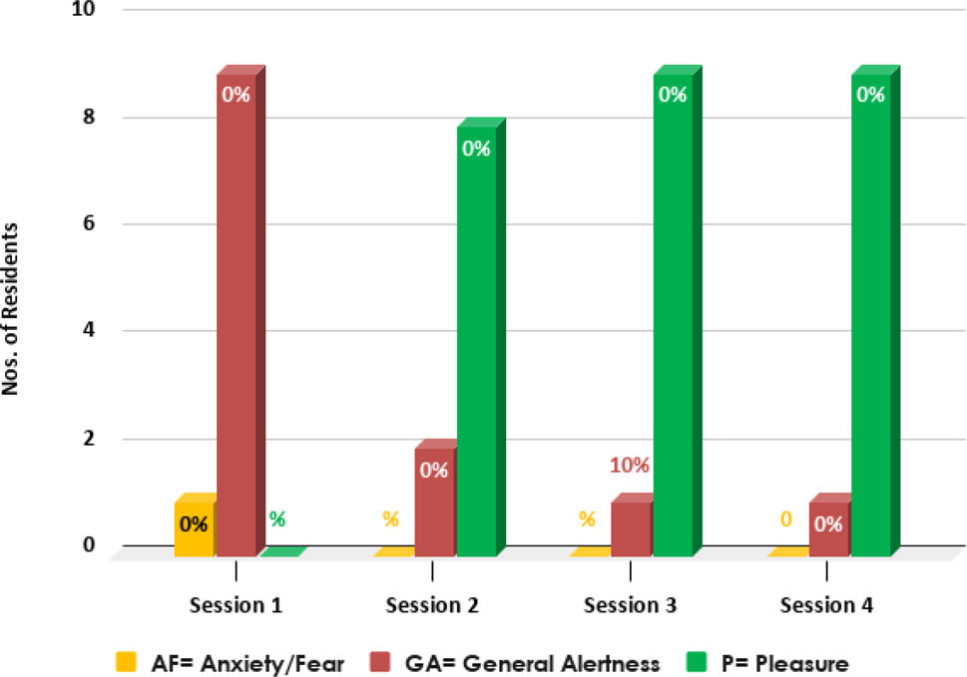
OERS Summary. Session one denotes the initial 10 sessions; Session two shows the subsequent 4 sessions; Session three indicates the next 5 sessions; Session four represents the final 10 sessions

Per the OERS tool, as seen in Fig. 11, the emotion ratings of the 10 baseline sessions were 10% AF and 90% GA, which progressively improved over the interaction programme as the residents’ emotions improved to 90% P, 10% GA, and 0% of AF.

## Conclusion and discussion

We placed Nadine at a nursing home and studied the effects of her presence in one-to-one interactions with the residents through different means. A psychotherapist performed the studies via two chosen methods: computer- and observation-based assessments.

Contrary to expectations and previous findings [112], Nadine’s presence was associated with more activity from the staff over time. A few factors can explain this increase in the activity of the care staff during resident–Nadine interactions. First, residents started to engage more with Nadine over period of time. For residents to initiate conversations with Nadine or continue the conversations they needed help from the staff in picking up the mic and holding it. Also, Nadine’s English and Mandarin modules were unfamiliar to the residents, requiring staff to frequently translate and explain Nadine “expressions” and language. Residents who only spoke the dialect depended on the staff for translation to “talk” to Nadine. However, when Nadine gave appropriate responses, residents were observed to respond proactively too. The residents interacted with Nadine, on average, half of each day, indicating a moderate interest in interacting with Nadine.

The residents interacted with Nadine on average a half of the days that she was there. This indicates moderate interest of theirs for interacting with her, which is to be expected from the residents of a home for the elderly. However, those who interacted with her more showed much less fear, indicating that they felt more relaxed when interacting with her. They also smiled slightly less, but this may be attributed to them just normally interacting with Nadine. This is also confirmed by the fact that they expressed neutral emotions more, which is in line with previous studies which showed more calmness after interacting with Paro [29] but also contradictory to studies which showed less neutral affect for residents interacting with Paro [30].

On average the residents conversed with Nadine for around 15 minutes. This is most likely the attention span that they are able to invest in the conversation with her. It may also be the limit of their interest in the interaction. With time, the residents became more and more accustomed to Nadine, as the conversations became longer over time. Furthermore, the residents showed less smiling and more neutral expressions with longer conversations. This is probably due to them finding Nadine less funny or mesmerizing over time, but more interesting as a conversation partner.

The findings indicate significant positive changes noted in the residents’ responses (well-being) across the interaction programme with Nadine. The key findings from the observational results suggest that residents responded positively to Nadine through increased positive emotional expression, quality of engagement, and engagement duration. These outcomes were partially supported by the numerical data from the computer- and observation-based methods, which showed no changes in positive emotions that were rather high throughout the assessment. Furthermore, disgust decreased with time. Additionally, residents’ positive emotions (like smile and neutral) overwhelmingly dominated negative ones (like fear, disgust, contempt, and anger); sadness was moderately expressed but still at a lesser degree than smile and neutral emotions.

Analytical findings using both the observational data and case studies were encouraging for human–robot interaction (HRI), suggesting the following:

- *Improved well-being for residents in nursing homes: * Residents in nursing homes appeared to accept and benefit from their interactions with Nadine, impacting their psychosocial, emotional, and cognitive needs. All our participants demonstrated responses that surpassed the usual nursing home activity engagement; they had opportunities to display the different domains of empathy that provided a sense of purpose and connection with the social robot. The residents also showed higher activity based on numerically increased optical flow, which is excellent for their motoric and social development. This type of engagement is often not encountered in nursing homes; it provides opportunities for residents to challenges their curiosity and try to understand the social robot, motivates them to find ways to connect with it, and instils in them the patience and nurturing spirit towards helping their companion. Eventually, there were noticeable improvements in the communication abilities for 90% of the residents, regardless of their cognitive abilities. Nadine instilled in the residents a sense of purpose and affection, allowing them to care for and nurture the robot.
- *The residents who interacted less frequently with Nadine were probably afraid of her: * Fear and the frequency of interactions with Nadine correlated negatively, indicating that the elderly who interacted less with her may have feared her. Despite her highly humanoid appearance and behaviour, some of the elderly still feared her, which is unfortunate and a missed opportunity for development. The elderly probably need more time and education to get used to Nadine and stop being afraid. However, the apparent positivity others derived from interacting with Nadine could have spread among the fearful residents over time.
- *Emotional expression Responses: * Those who interacted more with Nadine showed much less fear and an increasing level of affection for Nadine through their nonverbal and verbal communications, indicating that they felt more relaxed with her. These residents also smiled slightly less, possibly because they perceived their interaction with Nadine as purely normal. Their more frequent expression of neutral emotions supports this assumption, which is in line with observations from previous studies showing more calmness after interacting with Paro [29] However, our observation contradicts the view from another study with less neutral affect for residents interacting with Paro [30]. Still, our inclusion of new tests (presented above) that previous studies had not run should tilt the pendulum of advantage towards our findings.
- *Positive intervention for residents with cognitive impairment or dementia: * Residents with cognitive impairment and dementia appeared to improve their cognitive abilities as they interacted with Nadine. As seen in the eight residents with cognitive impairment and/or dementia, their communication and language abilities and mental processes improved significantly. Despite their cognitive impairment, their level of empathy appeared reasonably intact and may have compensated for their cognitive deficits and promoted appropriate responses and behaviours; this sparks exciting possible interventions where social robots could stimulate and promote the unaffected senses in persons with cognitive impairment.
- *Increased productivity by augmenting or reducing human resources: * Social robots can augment or reduce the staffing needed in nursing homes. This study featured the use of a social robot that was operational at fixed timing. Residents only interacted with Nadine at specific times. Despite the robot’s rigidity and inconsistent performance, the residents were very accommodating and affectionate and nurtured it.

Since Nadine has a humanoid appearance and the ability to communicate, read, and make facial expressions related to emotions, it is logical to assume that her presence can simultaneously improve the state of residents in nursing homes and reduce the burden on the nursing home staff. The realism of Nadine’s appearance and interactions is of paramount importance for her usage in human interactions, especially amongst the elderly. Because this group of citizens is least familiar with technology, this gap could be bridged using human-like robots. All the benefits of having people do a particular job could henceforth be combined with the benefits of using robots, which leads to the best possible outcomes for both users and organisations. That is why research on humanoid robots is so important and why it needs further development.

This study showed that using Nadine in a one-to-one interaction (video captured during all sessions^3^) setting could be beneficial to cognitively impaired residents in nursing homes, which is a step in the right direction. Future studies should continue investigating these issues and determining all the settings in which humanoid robots’ usage could be beneficial.

## Future research

We confirmed that using social robots can undoubtedly help the well-being of care home residents. However, much scientific work must still be conducted on and with these robots to ensure the continuity of users’ well-being daily: more research has to be conducted on speech recognition and understanding, the awareness of social robots, multi-party interaction, and the capability to move around and intervene with residents. The social robot should also combine multi-functions to grasp objects and bring them wherever the resident is.

A better speech recogniser must also be developed to understand the broken/soft speech spoken by the elderly. We observed that Nadine could not always answer the elderly correctly due to her inability to understand their speech, an issue not faced by healthy adults. In general, speech recognition systems are optimised to an average adult’s voice, with speech tending to exhibit a lower accuracy rate when recognising an older adult’s voice because of speech articulation and speaking style. The elderly’s speech patterns have a slower speech rate with lengthier inter-syllabic silence and slightly lower speech intelligibility. Therefore, the speech synthesiser must be modified to adapt speech output to make it easier for the elderly to understand them. Speech understanding significantly degrades with ageing, particularly in noisy environments. A robot should speak slower and have a higher volume with a low tone to help the elderly understand and interact effectively. The synthesiser must convey appropriate emotions while maintaining the speed of the robot speech. Age-related deficits in speech-in-noise understanding pose a significant problem for older adults. There is a need to develop a better and dedicated speech synthesiser for assistive robots that serve the purpose of companionship to the elderly.

Our experiments demonstrate the need for socially assistive robots to operate in environments with multiple users. The robot control systems that govern these multi-party interactions must be evaluated from technical and social standpoints(97). Social robots are becoming unstuck in more and more situations where they must interact with multiple users simultaneously. We believe that the multi-party interaction system will render robots more communicative, cooperative, intuitive, capable of meeting expectations, and, overall, able to make a better impression.

Our findings provide an excellent opportunity to develop a mobile social robot that increases accessibility and interaction opportunities. A roving social robot with humanoid features could support the existing staffing resources as it can generate optimal psychosocial, emotional, and cognitive responses without the constant presence of a human worker. At study sites, a roving robot could serve as an enhancement to the workforce by moving around to generate active and passive engagement from the residents within the daily activity area through conversations with and among the residents, providing video and audio reminiscence materials, and being an exciting and interesting object to behold.

## Footnotes

1 http://dlib.net/

2 https://github.com/open-mmlab/mmaction2

3 https://www.dropbox.com/sh/hln4fhnj15o8d95/AACWKdFDI9r5HDjuCQSlVXpRa?dl=0
